# Secondary breast surgery after initial partial mastectomy for breast cancer in Japan

**DOI:** 10.1007/s00595-026-03429-3

**Published:** 2026-07-20

**Authors:** Munetsugu Hirata, Yuko Kijima, Sam E. Wing, Peng-Lin Lin, Hisamitsu Zaha

**Affiliations:** 1 Breast Surgery Department, Fujita Health University Hospital, 1-98 Dengakugakubo, Kutsukake-cho, Toyoake, Aichi Japan; 2 Intuitive Surgical, 1020 Kifer Road, Sunnyvale, CA USA; 3 Nakagami Hospital, 610 Noborikawa, Okinawa, Japan

**Keywords:** Breast Neoplasms, Mastectomy, Segmental, Reoperation, Healthcare Costs

## Abstract

**Background:**

Partial mastectomy for breast cancer may be followed by unplanned secondary surgery due to adverse outcomes, such as the detection of positive margins or cancer recurrence, thus adding burden to patients, providers, and payers. We analyzed the prevalence of this outcome and its economic impact in Japan.

**Methods:**

We utilized the Medical Data Vision (MDV) Research Database to identify a cohort of women in Japan who underwent an index partial mastectomy between 2008 and 2023, following their healthcare encounters up to 5 years postoperatively, including any secondary partial or total mastectomies.

**Results:**

Overall, 49,659 patients with breast cancer underwent primary partial mastectomy, and 2,203 (4.4%) underwent a second surgery. Among these secondary procedures, approximately two-thirds were total mastectomies. The median five-year postoperative healthcare expenditure among patients with one primary partial mastectomy was ¥1.8 million, and ¥2.5 million among patients requiring more than one procedure. Secondary procedures were more common among younger patients, those with larger tumors, and those who did not receive neoadjuvant therapy.

**Conclusion:**

Partial mastectomy is an essential surgical treatment for early stage breast cancer in Japan. While secondary surgery rates are low compared with other countries, these procedures still have negative financial consequences and extend patients’ journeys to remission.

**Supplementary Information:**

The online version contains supplementary material available at https://doi.org/10.1007/s00595-026-03429-3.

## Introduction

In Japan, breast cancer is the most common cancer by age-standardized incidence rate, with 74 per 100,000 diagnoses per year [1]. Approximately 55% of patients are diagnosed with localized disease, and this proportion has increased relative to regional and distant disease since the 1990s [2]. The incidence rates of breast cancer have been increasing, with an annual average increase of almost 5%, the fastest rate of change among a study of 36 global nations [3]. The increase in incidence may be linked to multiple factors. Japanese breast screening rates are under 50%, [4] which is well behind Western countries with mammography rates of 70–80% [5]. Further, decreasing childbirth rates and higher obesity rates [6] may also partially explain this trend.

The primary therapeutic option for patients with early stage breast cancer is partial mastectomy (i.e., breast-conserving surgery), which removes a portion of the breast but leaves the majority of the tissue intact. Partial mastectomy has been strongly recommended for the local treatment of early stage cancer since the 2013 guidelines from the Japanese Breast Cancer Society [7]. Partial mastectomy rates rose through the 2000 s to comprise approximately 60% of all breast surgical cases by 2010. However, this trend has since reversed, and partial mastectomies are performed at a similar rate as total mastectomies [8]. While the 5-year overall survival and breast cancer-specific survival after partial mastectomy plus adjuvant radiotherapy are high (95% and 98%, respectively) [9], approximately one-quarter of the patients in Western countries may require additional unplanned therapeutic resections [10–13]. This may involve either repeat partial mastectomy or a subsequent total mastectomy to address issues such as detection of a positive surgical margin or cancer recurrence.

Until the 2010 s, the secondary surgery rates had historically approached 25% [10–13]. However, in 2013, the Society of Surgical Oncology and the American Society for Therapeutic Radiology and Oncology (SSO-ASTRO) released updated consensus guidelines to include “no ink on tumor” as the standard margin assessment for invasive tumors [14]. Research following this guideline update indicated a subsequent decrease in secondary surgery rates, falling to approximately 14% [15, 16]. Despite this overall reduction, additional surgeries still present a meaningful challenge to patients and providers. Although reoperations do not typically negatively impact survival [17], they are a source of clinical inefficiencies, excess costs, and reduced patient quality of life [18–20]. However, a key limitation of the existing literature on secondary surgery rates is that research has been conducted predominantly in Europe and the Americas. Limited research on these additional procedures or their economic impact has so far been published using Japanese data. This analysis aimed to better understand the frequency and financial implications of secondary surgeries after an index partial mastectomy for breast cancer treatment.

## Methods

### Materials

This retrospective analysis utilized the Medical Data Vision (MDV) database from 2008 to 2023. MDV is a Japanese healthcare claims database that captures nationwide encounter-level information on outpatient and inpatient visits. The database contains information on patient demographics, diagnoses, procedures, drug prescriptions, laboratory results, and medical costs. De-identified data were aggregated from participating healthcare facilities, representing 23% of acute care hospitals in Japan in 2022 [21]. MDV also reports hospital-level information, including bed size and cancer therapeutic facility status.

### Patients

**Fig. 1:**
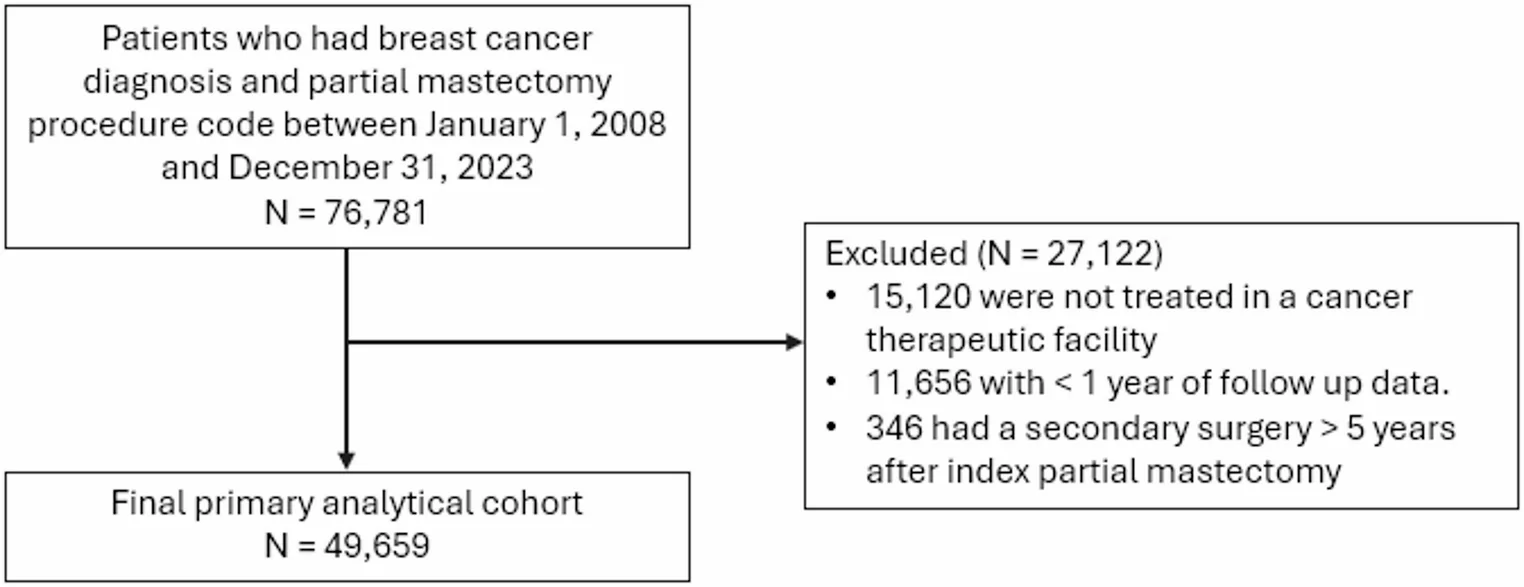
Patient inclusion and exclusion flow diagram

Eligible patients were identified from the MDV database using ICD-10 diagnosis and procedure codes, Kubun codes, and service dates. The included patients were women diagnosed with invasive breast cancer who underwent partial mastectomy between 2008 and 2023 at a cancer therapeutic facility and had at least 1 year of follow-up data available (Fig. 1). Follow-up was defined by the presence of any healthcare encounters, cancer-related or non-cancer-related, in the MDV database following the index partial mastectomy. During the post-index follow-up period, all healthcare encounters were evaluated to identify whether patients had undergone secondary surgery, which was classified as repeat partial mastectomy or subsequent total mastectomy based on procedure codes.

Patient and disease characteristics were obtained from the MDV database. Patient sex, age, smoking history, height, and weight were available in the discharge data. Height and weight were used to calculate and categorize BMI according to the Japan Society for the Study of Obesity definitions (e.g., underweight, < 18.5 kg/m^2^; normal, 18.5 to < 25 kg/m^2^; obese, ≥ 25 kg/m^2^). All patient diagnosis codes were compiled to calculate the Charlson Comorbidity Index (CCI), [22] which was categorized into 0, 1–2, 3–4, and ≥ 5 comorbidity count groupings. Cancer TNM staging was extracted and presented according to the individual T, N, and M statuses. Triple-negative, HER2-positive, lobular, and ductal breast cancer statuses were determined using diagnosis codes. Receipt of neoadjuvant therapy was assessed in the nine months preceding the index procedure, and adjuvant therapy was assessed in the 12 months following the index procedure, both identified via the Kubun code for chemotherapy and/or radiotherapy. Costs were calculated as the sum of all fee-for-service medical costs accrued during the relevant follow-up period after the index partial mastectomy.

### Statistical analysis

Secondary surgery rates were calculated based on the presence of a second oncologic breast surgery (i.e., partial or total mastectomy) within 5 years of the index partial mastectomy. These rates were compared by covariates, including patient, disease, and hospital characteristics, and balance was assessed for differences by secondary surgery status with the absolute standardized mean difference (ASMD); a covariate was considered meaningfully imbalanced when ASMD > 0.10 [23].

To compare downstream clinical and economic outcomes between patients with and without secondary surgery, we applied overlap weighting based on a propensity score model for secondary surgery [24]. In this study, overlap weighting was used to provide adjusted descriptive comparisons of medical expenditures rather than to estimate the causal effect of secondary surgery. Covariate balance after weighting was evaluated using ASMDs, which were < 0.10 for all covariates (Table 2). The covariates included sex, age, Charlson Comorbidity Index, BMI, TNM stage, neoadjuvant therapy receipt, TNBC status, HER2 status, hospital size, and follow-up duration. Overlap weighting was performed using the R package WeightIt (version 1.5.0) [25].

Total medical costs by secondary surgery status were assessed at 1-, 3-, and 5-years post-index, and group medians were tested using the Wilcoxon rank-sum test. Costs were standardized to 2023 values using Japanese consumer price index data from the World Bank [26]. Sensitivity analyses were performed to examine two subpopulations. First, a subset of patients whose second surgery occurred within three months of their index partial mastectomy. While no data for secondary surgical indications exist in this database (i.e., disease recurrence vs. detection of positive surgical margins), we hypothesize that re-excision of margins, but not disease recurrence, is more likely to be represented in this population subset. Second, the inclusion criteria of a minimum of one year of post-index follow-up were removed to assess any possible survivorship bias. All analyses were performed using R (version 4.1.3).

### Ethics

The MDV database deidentified and anonymized patient data, adhering to the Personal Information Protection Law [27] and has undergone an ethical review as part of its data-sharing agreements with participating hospitals. As the data were de-identified, informed consent was not required.

## Results

**Fig. 2:**
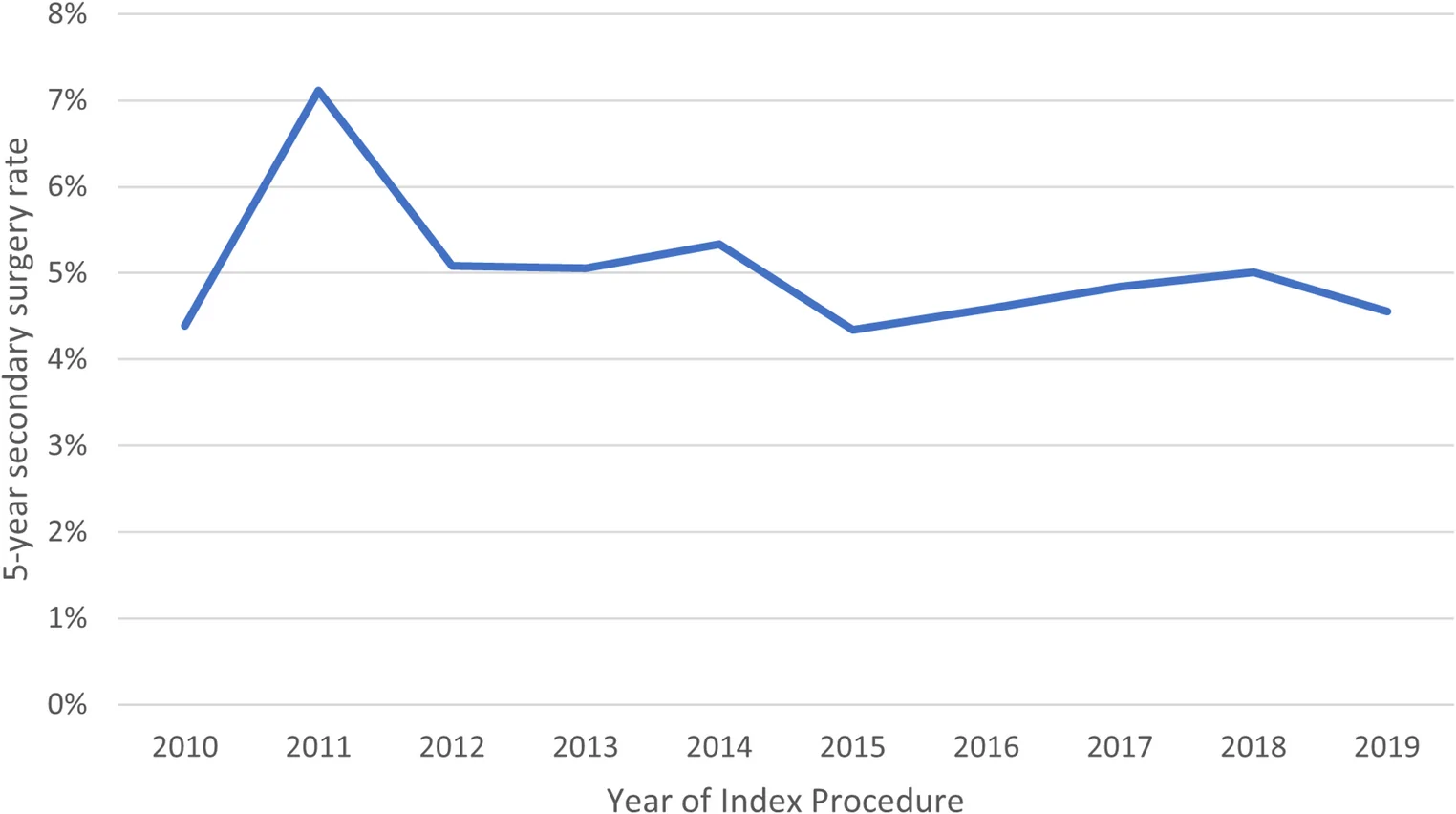
5-year secondary surgery rate

From 2008 to 2023, 49,659 eligible individuals were identified as having a breast cancer diagnosis and an index partial mastectomy. The number of partial mastectomy cases among eligible patients increased over time, from 914 in 2011 to 6,077 in 2021. Patients had an average of 4.6 (SD = 2.6) years of post-partial mastectomy follow-up period. During all available follow-ups, 2,549 patients underwent a second oncologic breast surgery. In the primary analysis, which defined secondary surgery as occurring within 5 years of index partial mastectomy, 1,476 (3.0%), 1,883 (3.8%), and 2,203 (4.4%) patients underwent secondary surgery by 1-, 3-, and 5- years, respectively. These secondary surgeries were predominantly mastectomies, with 1,449 (65.8%) of the 2,203 secondary surgeries within five years being total mastectomies (Table 1). Seventy patients (0.1%) underwent three or more total resections during the follow-up period. Throughout the study period, the 5-year secondary surgery rates remained largely stable (Fig. 2). The median time to the second procedure was 7.7 weeks after 1 year of follow-up (IQR: 5.1–11) and 10.9 weeks after 5 years of follow-up. (IQR: 6.3–93.0) Before overlap weighting, at 5 years post-index partial mastectomy, secondary surgery was associated with younger age (59.9 years vs. 56.1 years, ASMD = 0.41), carcinoma in situ tumors (Tis: 11.8% vs. 19.1% Tis, ASMD = 0.19), and TNBC (27.3% vs. 34.7%, ASMD = 0.16). After applying overlap weighting, the secondary surgery rates were balanced between the covariates (Table 2).

**Table 1:** Secondary surgery rates by follow-up period

	Secondary surgery rate	Second partial mastectomy rate
3-month (*n*, %)	1,224 (2.46%)	414 (33.8%)
1-year (n, %)	1,476 (2.97%)	473 (32.05%)
3-year (n, %)	1,883 (3.79%)	635 (33.70%)
5-year (n, %)	2,203 (4.44%)	754 (34.23%)
Ever (n, %)	2,549 (5.13%)	876 (34.37%)

**Table 2:** Baseline characteristics before and after applying overlap weighting

Characteristics	Before overlap weighting			After overlap weighting		
	No Secondary Surgery (*n* = 47,456)	Secondary Surgery (*n* = 2,203)	ASMD	No Secondary Surgery* (*n* = 39,813)	Secondary Surgery* (*n* = 2,202)	ASMD
**Sex**						
Female (n, %)	47,456 (100.0%)	2,203 (100.0%)	0.000	100.0%	100.0%	0.000
Male (n, %)	0 (0.0%)	0 (0.0%)	0.000	0.0%	0.0%	0.000
**Age (mean**,** SD)**	59.89 (12.8)	56.08 (13.4)	**0.412**	56.29 (12.4)	56.29 (13.5)	<0.001
**BMI classification†**						
Normal (n, %)	30,147 (63.53%)	1,422 (64.55%)	0.021	65.23%	64.44%	0.017
Underweight (n, %)	3,656 (7.70%)	212 (9.62%)	0.068	8.55%	9.58%	0.036
Obese (n, %)	13,653 (28.77%)	569 (25.83%)	0.066	26.22%	25.98%	0.006
**CCI (mean**,** SD)**	0.1 (0.7)	0.1 (0.6)	<0.001	0.1 (0.6)	0.1 (0.7)	<0.001
**CCI group**						
0 (n, %)	45,879 (96.68%)	2,160 (98.05%)	0.086	97.42%	98.00%	0.039
1~2 (n, %)	888 (1.87%)	21 (0.95%)	0.078	1.61%	0.95%	0.058
3~4 (n, %)	373 (0.79%)	9 (0.41%)	0.049	0.52%	0.41%	0.017
5~ (n, %)	316 (0.67%)	13 (0.59%)	0.010	0.45%	0.59%	0.020
**Smoking Index**						
Non-smoker (n, %)	38,003 (80.08%)	1,726 (78.35%)	0.043	78.78%	78.38%	0.010
~400 (n, %)	4,620 (9.74%)	245 (11.12%)	0.045	10.99%	11.04%	0.002
400~799	1,895 (3.99%)	85 (3.86)	0.007	4.12%	3.86%	0.013
800~ (n, %)	2,938 (6.19%)	147 (6.67%)	0.020	6.12%	6.72%	0.025
**Clinical T stage**						
T0 (n, %)	94 (0.20%)	9 (0.41%)	0.038	0.39%	0.41%	0.002
Tis (n, %)	5,608 (11.82%)	420 (19.06%)	0.202	18.61%	18.62%	<0.001
T1 (n, %)	31,013 (65.35%)	1,238 (56.20%)	0.188	56.67%	56.68%	<0.001
T2 (n, %)	9,971 (21.01%)	496 (22.51%)	0.036	22.54%	22.52%	<0.001
T3 (n, %)	334 (0.70%)	24 (1.09%)	0.041	1.06%	1.04%	0.002
T4 (n, %)	436 (0.92%)	16 (0.73%)	0.021	0.74%	0.73%	0.001
**Clinical N stage**						
N0 (n, %)	42,012 (88.53%)	1,950 (88.52%)	<0.001	88.54%	88.51%	0.001
N1 (n, %)	4705 (9.91%)	214 (9.71%)	0.007	9.73%	9.72%	<0.001
N2 (n, %)	430 (0.91%)	16 (0.73%)	0.020	0.73%	0.73%	0.001
N3 (n, %)	309 (0.65%)	23 (1.04%)	0.043	1.00%	1.00%	0.001
**Clinical M stage**						
M0 (n, %)	47,237 (99.54%)	2,187 (99.27%)	0.034	99.30%	99.27%	0.003
M1 (n, %)	219 (0.46%)	16 (0.73%)	0.034	0.70%	0.68%	0.003
**Neoadjuvant chemotherapy (n**,** %)**	4,881 (10.29%)	190 (8.62%)	0.057	8.72%	8.72%	<0.001
**Neoadjuvant radiotherapy (n**,** %)**	80 (0.17%)	0 (0%)	0.058	0.00%	0.00%	<0.001
**Lobular carcinoma (n**,** %)**	4,544 (9.58%)	256 (11.62%)	0.066	9.85%	11.63%	0.058
**Ductal carcinoma (n**,** %)**	61 (0.13%)	2 (0.09%)	0.011	0.11%	0.09%	0.005
TNBC* (n,** %)**	12,976 (27.34%)	765 (34.73%)	0.160	34.27%	34.29%	<0.001
**HER2 positive (n**,** %)**	36,134 (76.14%)	1,613 (73.22%)	0.067	73.30%	73.30%	<0.001
**Hospital bed size**						
~200 (n, %)	468 (0.99%)	22 (1.00%)	0.001	0.99%	1.00%	<0.001
200~499 (n, %)	21,338 (44.96%)	944 (42.85%)	0.043	42.94%	42.92%	<0.001
500~ (n, %)	25,650 (43.69%)	1,237 (56.15%)	0.042	56.06%	56.04%	<0.001

**ASMD**: absolute standardized mean difference; **SD**: standard deviation; **BMI**: body mass index; **CCI**: Charlson comorbidity index; **TNBC**: triple-negative breast cancer; **HER2**: human epidermal growth factor receptor 2; **IQR**: interquartile range.

†The Japan Society for the Study of Obesity (JASSO) defines BMI classifications as follows: underweight, <18.5 kg/m^2^; normal, 18.5 to <25 kg/m^2^; obese, ≥25 kg/m^2^

*Approximate effective sample size

Most patients received adjuvant therapy, with 39,877 (80.3%) receiving postoperative radiotherapy and/or chemotherapy. Patients who did not receive adjuvant therapy had higher secondary surgery rates than the main cohort, including after overlap weighting (*p* < 0.001) (Table 3). After overlap weighting, pre-operative neoadjuvant chemotherapy did not differ between groups, as it was administered in 8.4% of patients with a single partial mastectomy vs. 8.7% of patients undergoing secondary surgery (ASMD = 0.01). Similarly, neoadjuvant radiotherapy did not differ between the groups (ASMD < 0.001) (Table 2).

**Table 3:** Clinical outcomes before and after applying overlap weighting

Characteristics	Before overlap weighting			After overlap weighting		
	No Secondary Surgery (*n* = 47,456)	Secondary Surgery (*n* = 2,203)	*p*-value	No Secondary Surgery* (*n* = 39,813)	Secondary Surgery* (*n* = 2,202)	*p*-value
**Recurrence (n, %)**						
**3-month (n**,** %)**	**353 (0.74%)**	**27 (2.21%)**	**<0.001**	**0.71%**	**2.21%**	**<0.001**
1-year (n, %)	353 (0.74%)	33 (2.24%)	**<0.001**	0.72%	2.24%	**<0.001**
3-year (n, %)	353 (0.74%)	42 (2.23%)	**<0.001**	0.78%	2.23%	**<0.001**
5-year (n, %)	353 (0.74%)	46 (2.09%)	**<0.001**	0.79%	2.09%	**<0.001**
Ever^**†**^ (n, %)	353 (0.74%)	47 (1.84%)	**<0.001**	0.80%	1.84%	**<0.001**
**Adjuvant chemotherapy (n**,** %)**	11,397 (24.02%)	610 (27.69%)	**<0.001**	30.55%	27.39%	**0.002**
**Adjuvant radiotherapy (n**,** %)**	36,336 (76.57%)	902 (40.94%)	**<0.001**	78.19%	40.88%	**<0.001**

*Approximate effective sample size

†The max tracking year is 14

Compared with patients who underwent a single partial mastectomy, those who underwent secondary surgery had increased 1-, 3-, and 5-year median healthcare costs of ¥365,678, ¥649,926, and ¥900,572 (*p* < 0.001). These differences remained stable after overlap weighting. At 5 years post-index partial mastectomy, a patient with three total procedures generated costs 85% higher than patients with a single partial mastectomy (¥3,370,538 vs. ¥1,825,410, *p* < 0.001). The costs for patients undergoing secondary surgery were higher among those who did not receive adjuvant treatment than in the overall cohort. At 5 years post-index, patients who underwent two total surgeries and adjuvant therapy (*n* = 1,216) had a median medical cost of ¥3,303,242 vs. ¥1,953,813 (*p* < 0.001) for patients with two total surgeries but no adjuvant therapy (*n* = 943).

Two sensitivity analyses were conducted using alternative follow-up times. First, only patients who underwent secondary surgeries in the first three months after the index partial mastectomy were assessed. The secondary surgery rate was 2.5% (Table S1), accounting for over half of the secondary surgeries across the 5-year follow-up period among patients in the main cohort. The second sensitivity analysis removed the one-year minimum inclusion criteria to assess survivorship bias (Table S2). The 1-, 3-, and 5-year secondary surgery rates remained similar to those of the main analysis, although very modestly attenuated (2.9%, 3.5%, and 4.1%, respectively).

## Discussion

The secondary surgery rates after partial mastectomy for the treatment of breast cancer in Japan are low compared with those in much of the Western world, ranging from 4.5% to 9.8%, depending on the receipt of adjuvant therapy. Most secondary procedures are performed because of positive margins [13], with approximately half being ink on tumor [12]. These rates are slowly decreasing over time; the pace of decrease may reflect only a gradual adoption of the updated partial mastectomy guidelines published in 2013. [28] While MDV data do not have sufficient information to determine the exact indication of the secondary procedure, over half of these secondary surgeries occurred within 3 months of the index partial mastectomy. Considering the limitations of the database, we cautiously posit that this finding may suggest that re-excision of margins could be a key cause of these returns to the operating room.

We hypothesized that, based on previous publications, surgical practices adopted in Japan may help minimize the incidence of additional procedures, especially compared to Western countries. First, Asian surgeons tend to adopt a more cautious oncologic approach, resecting more tissue than the standard in many Western countries [29, 30]. Second, intraoperative pathological margin assessment is guideline-endorsed in Japan [31], potentially reducing the need for these secondary surgeries, but it is not universally explicitly mandated by Western surgical societies [32, 33]. Third, surgeons at Japanese hospitals are twice as likely to conduct cylindrical excisions compared to international institutions [34]. Such oncoplastic resections may offer improved margin control [35] relative to conventional partial mastectomies. Finally, preoperative breast MRI is frequently used and is guideline-endorsed in Japan [36] to determine the eligibility for breast-conserving surgery and may further reduce the risk of additional surgeries. In contrast, these MRIs are not routinely recommended in the United States [37]. While these factors could not be directly tested using the MDV dataset, they provide possible insight into the global variation in the secondary surgery rates.

Unplanned, repeated breast surgery can create an additional burden in the treatment of breast cancer, especially for patients and the healthcare system. These secondary surgeries can induce a large financial burden; in the United States, an estimated $417 million USD of excess healthcare spending is generated each year due to the impacts of secondary procedures [20]. While Japan’s secondary surgery rate is markedly lower than current estimates in Western countries, these procedures could be associated with meaningful excess costs. Given that each secondary procedure adds a median of ¥900,572 in 5-year postoperative costs and that MDV represents approximately 23% of acute care centers in Japan, there are approximately 640 secondary cases each year, leading to ¥576 million excess costs per year associated with repeat breast surgery in Japan. Additionally, healthcare workforce challenges, such as the current surgeon shortage reported by over half of Japan’s teaching hospitals [38], mean that efficiency and limiting secondary surgeries are critical to both operational and financial stakeholders.

### Strengths and Limitations

This study has several limitations. First, patients are lost to follow-up if they seek care at a different hospital than the one where they received their index partial mastectomy. Second, these data were obtained from a claims database that was not originally designed for primary research. Some relevant data may be missing as they may not have been relevant for reimbursement purposes 21, which includes relevant disease and clinical characteristics such as margin status, pathological subtype details, and recurrence timing. As such, these analyses should be considered descriptive and not causal. There is a risk of survivorship bias by requiring patients to have a minimum of 1-year follow-up; however, the impact of excluding those patients with under 1-year follow-up was very small in magnitude. Finally, MDV’s coverage is focused on acute care facilities and may underrepresent smaller and lower-acuity centers, which may lead to an overrepresentation of more complex cases. The strengths of this study include good long-term follow-up (mean follow-up = 4.6 years), allowing for the description of treatments and costs attributable to cancer care, such as surgical interventions, adjuvant treatment, reconstruction, and other follow-up care. Additionally, MDV is a large database that is representative of the Japanese population.

## Conclusion

Although secondary surgeries after an index partial mastectomy can add to the financial burden of treating breast cancer, Japan has achieved rates below those of other countries, especially those in the West. The continued implementation of best practices and innovations in breast surgical techniques can aid in the sustained suppression of additional procedures.

## Supplementary Information

Below is the link to the electronic supplementary material.

Supplementary Material 1
