# Supplementary material for: Secondary breast surgery after initial partial mastectomy for breast cancer in Japan
Source: Surg Today. 2026 Jul 20;56(11):2797–804. doi: 10.1007/s00595-026-03429-3 (PMC13722626; doi:10.1007/s00595-026-03429-3)
Supplement: Supplementary file 1 [file 595_2026_3429_MOESM1_ESM.docx]

**Table S1. Baseline characteristics before and after applying overlap weighting within 3 months of the index surgery.**

|  | **Before overlap weighting** | | | **After overlap weighting** | | |
| --- | --- | --- | --- | --- | --- | --- |
| **Characteristics** | **No Secondary Surgery**  **(n = 47456)** | **Secondary Surgery**  **(n = 1224)** | **ASMD** | **No Secondary Surgery***  **(n = 39915)** | **Repeat Secondary Surgery***  **(n = 1223)** | **ASMD** |
| **Sex** |  |  |  |  |  |  |
| Female (n, %) | 47456 (100%) | 1224 (100%) | 0.000 | 100.0% | 100.0% | 0.000 |
| Male (n, %) | 0 (0.0%) | 0 (0.0%) | 0.000 | 0.0% | 0.0% | 0.000 |
| **Age (mean, SD)** | 59.89 (12.8) | 54.86 (12.1) | 0.404 | 56.17 (12.5) | 55.05 (12.1) | 0.091 |
| **BMI classification****†** |  |  |  |  |  |  |
| Normal (n, %) | 30147 (63.53%) | 784 (64.05%) | 0.011 | 64.99% | 64.02% | 0.02 |
| Underweight (n, %) | 3656 (7.7%) | 114 (9.31%) | 0.058 | 8.43% | 9.24% | 0.029 |
| Obese (n, %) | 13653 (28.77%) | 326 (26.63%) | 0.048 | 26.58% | 26.74% | 0.004 |
| **CCI (mean, SD)** | 0.1 (0.7) | 0 (0.5) | 0.008 | 0.1 (0.6) | 0.1 (0.5) | < 0.001 |
| **CCI group** |  |  |  |  |  |  |
| 0 (n, %) | 45879 (96.68%) | 1208 (98.69%) | 0.134 | 97.48% | 98.61% | 0.082 |
| 1~2 (n, %) | 888 (1.87%) | 21 (0.95%) | 0.078 | 1.59% | 0.65% | 0.089 |
| 3~4 (n, %) | 373 (0.79%) | 9 (0.41%) | 0.049 | 0.5% | 0.41% | 0.013 |
| 5~ (n, %) | 316 (0.67%) | 13 (0.59%) | 0.010 | 0.43% | 0.33% | 0.017 |
| **Smoking Index** |  |  |  |  |  |  |
| Non-smoker (n, %) | 38003 (80.08%) | 960 (78.43%) | 0.041 | 78.8% | 78.5% | 0.007 |
| ~400 (n, %) | 4620 (9.74%) | 141 (11.52%) | 0.058 | 11.05% | 11.45% | 0.013 |
| 400~799 | 1895 (3.99%) | 41 (3.35%) | 0.034 | 4.09% | 3.35% | 0.039 |
| 800~ (n, %) | 2938 (6.19%) | 82 (6.7%) | 0.021 | 6.07% | 6.7% | 0.026 |
| **Clinical T stage** |  |  |  |  |  |  |
| T0 (n, %) | 94 (0.2%) | 4 (0.33%) | 0.025 | 0.3% | 0.33% | 0.005 |
| Tis (n, %) | 5608 (11.82%) | 216 (17.65%) | 0.165 | 18.37% | 17.25% | 0.029 |
| T1 (n, %) | 31013 (65.35%) | 727 (59.4%) | 0.123 | 57.07% | 59.93% | 0.058 |
| T2 (n, %) | 9971 (21.01%) | 256 (20.92%) | 0.002 | 22.6% | 20.85% | 0.042 |
| T3 (n, %) | 334 (0.7%) | 15 (1.23%) | 0.053 | 0.99% | 1.23% | 0.022 |
| T4 (n, %) | 436 (0.92%) | 6 (0.49%) | 0.051 | 0.67% | 0.49% | 0.023 |
| **Clinical N stage** |  |  |  |  |  |  |
| N0 (n, %) | 42012 (88.53%) | 1100 (89.87%) | 0.043 | 88.33% | 90.02% | 0.055 |
| N1 (n, %) | 4705 (9.91%) | 108 (8.82%) | 0.037 | 10.09% | 8.75% | 0.046 |
| N2 (n, %) | 430 (0.91%) | 8 (0.65%) | 0.029 | 0.78% | 0.65% | 0.015 |
| N3 (n, %) | 309 (0.65%) | 8 (0.65%) | <0.001 | 0.81% | 0.65% | 0.018 |
| **Clinical M stage** |  |  |  |  |  |  |
| M0 (n, %) | 47237 (99.54%) | 1219 (99.59%) | 0.008 | 99.28% | 99.67% | 0.054 |
| M1 (n, %) | 219 (0.46%) | 5 (0.41%) | 0.008 | 0.72% | 0.41% | 0.041 |
| **Neoadjuvant chemotherapy (n, %)** | 4881 (10.29%) | 68 (5.56%) | 0.176 | 8.52% | 5.64% | 0.112 |
| **Neoadjuvant radiotherapy (n, %)** | 80 (0.17%) | 0 (0%) | 0.058 | 0.00% | 0.00% | <0.001 |
| **Lobular carcinoma (n, %)** | 4544 (9.58%) | 133 (10.87%) | 0.043 | 9.58% | 10.87% | 0.043 |
| **Ductal carcinoma (n, %)** | 61 (0.13%) | 1 (0.08%) | 0.014 | 0.11% | 0.08% | 0.009 |
| **TNBC* (n, %)** | 12976 (27.34%) | 355 (29%) | 0.037 | 28.86% | 27.06% | 0.04 |
| **HER2 positive (n, %)** | 36134 (76.14%) | 941 (76.88%) | 0.017 | 66.89% | 75.63% | 0.194 |
| **Hospital bed size** |  |  |  |  |  |  |
| ~200 (n, %) | 468 (0.99%) | 14 (1.14%) | 0.015 | 0.87% | 1.14% | 0.027 |
| 200~499 (n, %) | 21338 (44.96%) | 531 (43.38%) | 0.032 | 43.43% | 43.5% | 0.001 |
| 500~ (n, %) | 25650 (54.05%) | 679 (55.47%) | 0.029 | 55.7% | 55.36% | 0.007 |
| **ASMD**: absolute standardized mean difference; **SD**: standard deviation; **BMI**: body mass index; **CCI**: Charlson comorbidity index; **TNBC**: triple-negative breast cancer; **HER2**: human epidermal growth factor receptor 2; **IQR**: interquartile range.  †The Japan Society for the Study of Obesity (JASSO) defines BMI classifications as follows: **underweight**, <18.5 kg/m^2^; **normal**, 18.5 to <25 kg/m^2^; **obese**, ≥25 kg/m^2^.  *Approximate effective sample size. | | | | | | |

**Table S2. Secondary surgery rate with no minimum follow up time required**

|  | Secondary surgery rate | Second partial mastectomy rate |
| --- | --- | --- |
| 3-month (n, %) | 1462 (2.48%) | 517 (35.4%) |
| 1-year (n, %) | 1757 (2.87%) | 591 (33.6%) |
| 3-year (n, %) | 2164 (3.53%) | 753 (34.8%) |
| 5-year (n, %) | 2484 (4.05%) | 872 (35.1%) |
| Ever (n, %) | 2830 (4.62%) | 994 (35.1%) |
